# Demographic, health, and behaviors profile of Saudi Arabia’s aging population 2022–2023

**DOI:** 10.3389/fragi.2025.1491146

**Published:** 2025-02-26

**Authors:** Nasser F. Bindhim, Mohammed Senitan, Madhawi N. Almutairi, Leen S. Alhadlaq, Sundus A. Alnajem, Maryam Ali Alfaifi, Nora A. Althumiri

**Affiliations:** ^1^ Informed Decision Making (IDM), Riyadh, Saudi Arabia; ^2^ Sharik Association for Research and Studies, Riyadh, Saudi Arabia; ^3^ Department of Public Health, College of Health Sciences, Saudi Electronic University, Riyadh, Saudi Arabia; ^4^ Medical Cities Program, Ministry of Interior, Riyadh, Saudi Arabia; ^5^ College of Medicine, King Saud University, Riyadh, Saudi Arabia

**Keywords:** aging population, multimorbidity, elderly health, Saudi Arabia, mental health, lifestyle behaviors

## Abstract

**Background:**

The population aged 60 years and older in Saudi Arabia is rapidly increasing, leading to concerns regarding their health, socioeconomic status, and lifestyle behaviors. Aging is associated with a higher risk of chronic diseases, multimorbidity, and mental health issues, which can significantly affect the quality of life. However, national data on older people in Saudi Arabia remain limited.

**Aim:**

This study aims to profile older people in Saudi Arabia during the years 2022–2023, focusing on their demographic characteristics, socioeconomic status, health conditions, and lifestyle behaviors.

**Methods:**

Data were drawn from the Sharik Health Indicators Surveillance System (SHISS) 2022–2023, a nationwide cross-sectional survey conducted through phone interviews. The final analysis included 2,702 participants aged 60 years and older. Descriptive statistics were employed to summarize demographic, health, and behavioral data.

**Results:**

The study revealed that over half (52%) of the participants had two or more chronic conditions, with hypertension, hypercholesterolemia, and type 2 diabetes being the most common. Mental health assessments indicated that 17.7% of older people were at risk of depression, and another 17.7% were at risk of anxiety. Additionally, the study found low adherence to healthy behaviors, with only 11.1% meeting the recommended fruit and vegetable intake and 20.1% engaging in sufficient physical activity. Furthermore, 67% of older people were classified as overweight or obese.

**Conclusion:**

Older people in Saudi Arabia face significant health challenges, including high rates of multimorbidity, mental health risks, and poor lifestyle behaviors. These findings highlight the urgent need for targeted health interventions and educational programs tailored to older people, aiming to improve their quality of life and contribute to the national goals outlined in Saudi Arabia’s Vision 2030.

## 1 Introduction

According to the World Health Organization (WHO) and Saudi national laws, the population aged 60 years and older is defined as older people (World Health Organization, 2024; Abusaaq, 2015). The WHO estimates that the proportion of the global population aged 60 years and older will nearly double, increasing from 12% to 22% between 2015 and 2050 (World Health Organization, 2022). Moreover, in 2020, the number of people aged 60 years and older surpassed the number of children younger than 5 years (World Health Organization, 2022). In Saudi Arabia, the official percentage of the older population (60+) ranged from 5.59% to 6.9% of the total population between 2020 and 2022 (General Authority for Statistics, 2024; Abusaaq, 2015).

Aging increases the risk of chronic diseases and multimorbidity, including conditions such as dementia, heart disease, type 2 diabetes, arthritis, and cancers (Centers for Disease Control and Prevention, 2024b). A recently published article analyzing data from 126 peer-reviewed studies, which included nearly 15.4 million people worldwide, found that more than half (51.0%) of the global adult population aged 60 years and older had multimorbidity conditions (Chowdhury et al., 2023). In the United States, multimorbidity was reported in 62% of those aged 65–74 years and in 81.5% of those aged 85 years and older (Salive, 2013).

In Saudi Arabia, there is no national population-level data on the prevalence of chronic diseases or multimorbidity in older people. However, a study that examined the prevalence of chronic diseases among individuals aged 65 years and older in a single hospital found that, out of 5,874 patients, 58.1% had hypertension, 48.6% had diabetes mellitus, 4.9% had asthma, 27.5% had cardiac diseases, and 14.6% had lung diseases (Alsuwaidan et al., 2021).

The availability of recent, high-quality data on the health, behavioral, and mental factors affecting older people living in Saudi Arabia is essential. Such information provides valuable insights for decision-makers to plan and implement interventions that address current demands and strategic future needs. Furthermore, Saudi Arabia’s ambitious Vision 2030 includes a strategic goal to increase the average life expectancy from 74 years in 2016 to 80 years by 2030 (Saudi Vision, 2030, 2016). Therefore, understanding the current status of the older population is crucial for identifying gaps that require focused attention and improvement. The primary aim of this study is to provide a comprehensive descriptive profile of the older population in Saudi Arabia during 2022–2023. By examining their demographic characteristics, socioeconomic status, health conditions, and lifestyle behaviors, this study seeks to establish foundational baseline data that can inform policy development and guide future research. Given the limited national data currently available, this descriptive approach is critical to addressing knowledge gaps and supporting evidence-based strategies to improve the quality of life and health outcomes for older people in Saudi Arabia.

## 2 Materials and methods

### 2.1 Design

This research made use of secondary data obtained from the Sharik Health Indicators Surveillance System (SHISS) for the years 2022–2023. SHISS represents a comprehensive, nationwide cross-sectional survey conducted via phone interviews, encompassing all 13 administrative regions across Saudi Arabia (BinDhim et al., 2021).

### 2.2 Participant selection and sample size determination

The SHISS utilized a proportional quota sampling method to achieve a balanced distribution of participants, ensuring representation across all 13 administrative regions of Saudi Arabia. The sample was stratified by age and gender, with participants divided into two age groups (18–36 and 37+), reflecting the median age of 36 years in Saudi Arabia. Although the median age of participants in the broader SHISS dataset is 36 years, this study specifically focused on individuals aged 60 years and above. Data for this subgroup were extracted from the overall dataset, yielding 1,711 participants in 2022 and 991 participants in 2023, for a total of 2,702 older adults. This represents approximately 6% of the sample, aligning with the proportion of older adults in Saudi Arabia’s population (5.59%–6.9%).

The sample size for the SHISS was determined to ensure adequate statistical power for both regional comparisons and sampling quotas. It was calculated based on a medium effect size of around 0.25, with an 80% power and a 95% confidence level. To meet these requirements, each quota needed a minimum of 134 participants, resulting in 536 participants per region and a total of 6,968 participants per quarterly wave. The quota sampling process was fully automated and managed by ZdataCloud, eliminating the need for human intervention (zDataCloud, 2024). Once a quota was fulfilled, individuals with similar characteristics were excluded from further participation in the study.

### 2.3 Participant selection and recruitment process

Four Recruitment efforts were focused on adults (18 years and above) who only speak Arabic and are currently living in Saudi Arabia. Potential participants were selected using randomly generated phone numbers from the Sharik Association for Health Research database, which contains over 230,000 registered individuals across the 13 regions of Saudi Arabia. These participants had previously indicated their willingness to be involved in future research and had given their consent to be contacted. Trained interviewers conducted phone interviews, each lasting approximately 4–6 min. Participants were called up to three times; if no response was received, a new phone number with similar demographic characteristics was selected from the database. This process was repeated until the required quota was reached, at which point the recruitment process was automatically concluded (BinDhim et al., 2021).

While the phone interview methodology ensured broad geographic coverage, it may have introduced selection bias by excluding those without phone access or willingness to participate. Additionally, the reliance on self-reported data could result in reporting bias, particularly for sensitive topics like mental health and lifestyle behaviors. To mitigate these biases, efforts were made to ensure diverse demographic representation, including regional, age, and gender stratification through a proportional quota sampling method. Additionally, the survey instruments were designed following validated protocols to encourage honest and accurate responses while minimizing respondent discomfort.

The SHISS utilized the ZdataCloud research data collection system to manage and record data, incorporating modules for eligibility and sampling to ensure accurate sample distribution and minimize human-related bias. Upon receiving consent to participate, interviewers assessed participants’ eligibility based on the specified quota criteria. Only complete responses were included in the database. The data were coded and stored within the ZdataCloud system, which also allowed for monitoring the quality of data recording by linking it to individual data collectors. Occasionally, as the ZdataCloud system approached the completion of the targeted sample size, simultaneous phone call attempts led to multiple participants passing the eligibility process, resulting in a slight increase in sample size beyond the target for some quotas.

### 2.4 Questionnaire development and validation process

**FIGURE 1:**
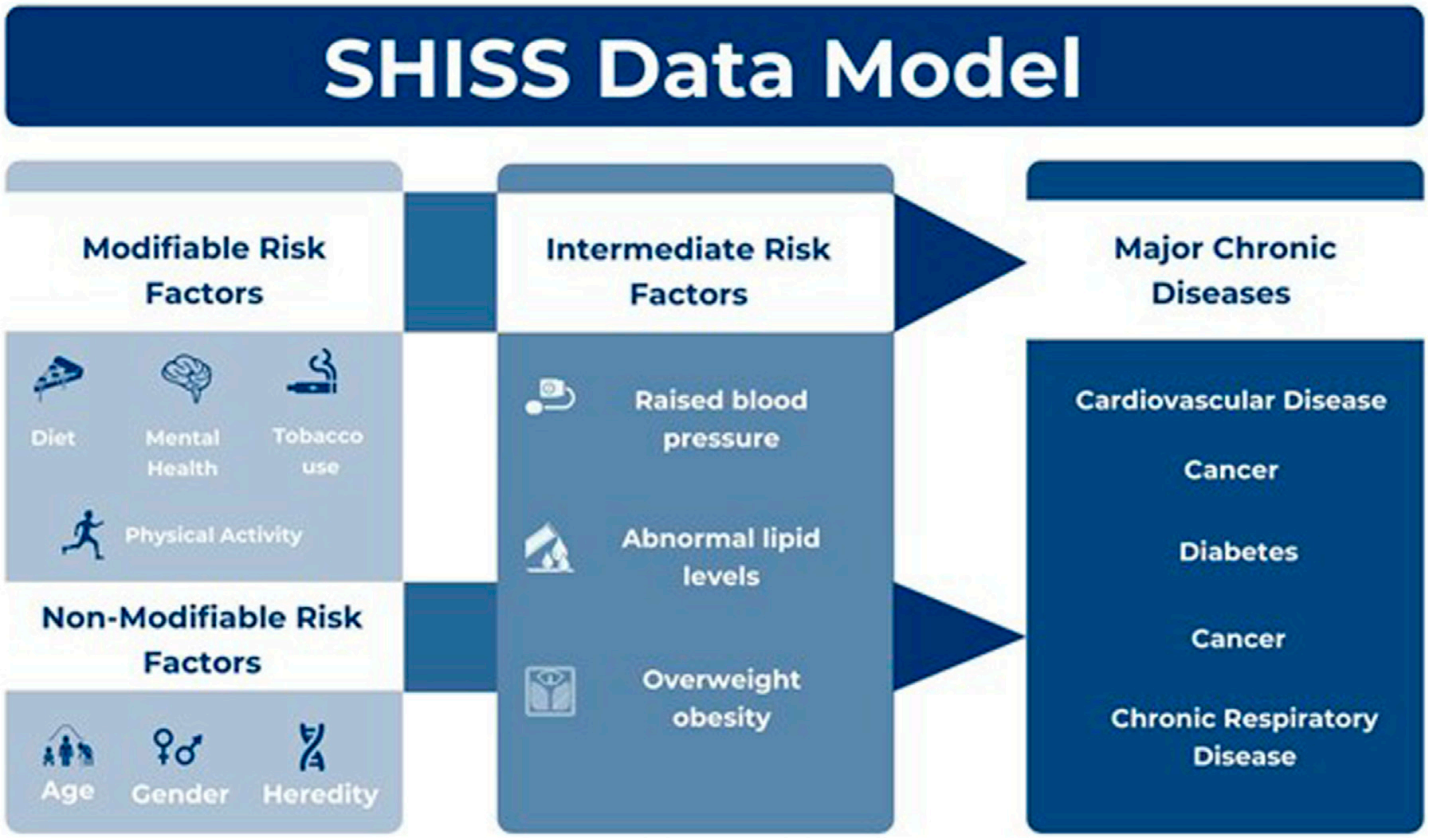
Sharik health indicators surveillance system (SHISS) (BinDhim et al., 2021)

The questionnaire design was guided by globally recognized frameworks, including the World Health Organization (WHO) and Centers for Disease Control and Prevention (CDC), ensuring the selection of indicators relevant to aging populations. These indicators included chronic diseases, intermediate risk factors, and behavioral factors such as dietary habits, physical activity, and tobacco use. Obesity was assessed using the body mass index (BMI), calculated from self-reported height and weight (Centers for Disease Control and Prevention, 2024a; World Health Organization, 2023). The SHISS data model (Figure 1) encompasses a range of behavioral risk factors, including dietary habits, physical activity levels, and the use of tobacco products, such as cigarettes, water pipes, and e-cigarettes (BinDhim et al., 2021). Intermediate risk factors, including hypertension, hypercholesterolemia, and obesity, were also documented, with obesity being assessed using the body mass index (BMI), calculated from participants’ self-reported height and weight (Centers for Disease Control and Prevention, 2022).

The questionnaire further explored the presence of major chronic diseases for which participants were currently receiving treatment, such as type 2 diabetes, heart disease, stroke, cancer, and chronic respiratory conditions (Centers for Disease Control and Prevention, 2022). For these chronic diseases and intermediate risk factors, participants were asked if they had been diagnosed by a physician (e.g., with hypertension) and whether they were currently receiving treatment. A “Yes” response to both questions led to the recording of the condition. Additionally, any diagnosed genetic diseases were noted as nonmodifiable risk factors.

The selection of indicators was grounded in evidence from international frameworks and previous studies highlighting their relevance to aging populations. For example, chronic disease prevalence and multimorbidity are emphasized in global studies on aging due to their significant impact on healthcare needs and quality of life (Chowdhury et al., 2023; Salive, 2013). Behavioral indicators, such as physical activity and dietary patterns, are critical for understanding modifiable risk factors that can inform public health interventions (Centers for Disease Control and Prevention, 2024a). This theoretical grounding underscores the importance of the selected indicators in providing a comprehensive profile of Saudi Arabia’s older population.

In 2021, the SHISS questionnaire was expanded to capture additional variables, including income, education level, and mental health assessments. For mental health, tools such as the validity of the Patient Health Questionnaire 2 (PHQ‐2) in identifying major depression in older people and the Generalized Anxiety Disorder-2 (GAD-2) and GAD-7 in a primary care setting for anxiety screening were introduced.

### 2.5 Data extraction, retrieval, and compilation

We classified older participants as those aged 60 and above, in accordance with the definitions provided by the United Nations and the World Health Organization. For this study, we specifically extracted data from participants in the SHISS 2022 and 2023 surveys. The total number of participants in SHISS was 27,204 in 2022 and 13,913 in 2023. After applying the age filter, we identified 1,711 participants in 2022 and 991 participants in 2023, resulting in a final sample size of 2,702 older individuals.

### 2.6 Statistical analysis

Descriptive statistics were used to summarize the prevalence data. Quantitative variables are presented as mean and standard deviation (SD) for normally distributed data, or as median and range for non-normally distributed data. Categorical variables were reported as percentages.

The cross-sectional nature of the dataset limits causal inference but offers valuable insights into population-level patterns. The use of descriptive statistics aligns with the study’s primary objective of providing a detailed demographic, health, and behavioral profile of Saudi Arabia’s aging population. Given the cross-sectional design of the dataset, our analysis focuses on summarizing patterns and prevalence without attempting to establish causal relationships.

The use of an electronic data collection system, equipped with built-in submission checks, ensured that no data were missing. Furthermore, ZdataCloud’s data integrity protocols effectively prevented the entry of invalid data. The study’s findings were documented following the Strengthening the Reporting of Observational Studies in Epidemiology (STROBE) guidelines for cross-sectional research (Vandenbroucke et al., 2007).

### 2.7 Ethical compliance and approval

This research project received approval from the ethics committee of the Sharik Association for Health Research (Approval No. 2021–2) and was conducted in accordance with national research ethics guidelines. Verbal consent from participants was secured during phone interviews and recorded within the data collection system, although no audio recordings were taken. The study was conducted in full adherence to the principles of the Declaration of Helsinki (World Medical Association, 2022).

## 3 Results

Table 1 demonstrates the demographic characteristics of the 2,702 participants aged 60 years and older included in the study. These participants represent all 13 administrative regions of Saudi Arabia, constituting 6.6% of the total SHISS sample during the same period. The majority of participants were male (59.8%) with a mean age of 66.66 years (range: 60–90 years). The highest representation was from Riyadh and Qassim (9.8% each), while Tabuk had the lowest representation (5.2%). Educational attainment varied, with 23.8% having less than elementary education and 19.3% holding a bachelor’s degree. Nearly 26.7% reported an income below 5,000 SR, and 18.8% had no stable income.

**TABLE 1:** Participant demographical characteristics.

Characteristics	Total (%)
Gender	
Male	1,617 (59.8)
Female	1,085 (40.2)
Age mean (min - max)	66.66 (60–90)
Regions	
Asir	195 (7.2)
Baha	211 (7.8)
Eastern region	189 (7.0)
Hail	229 (8.5)
Jazan	216 (8.0)
Al Jouf	198 (7.3)
Madinah	198 (7.3)
Makkah	189 (7.0)
Najran	183 (6.8)
Northern border	250 (9.3)
Qassim	215 (9.8)
Riyadh	265 (9.8)
Tabuk	140 (5.2)
Educational Level	
Less than elementary school	644 (23.8)
Just completed elementary school	361 (13.4)
Just completed intermediate school	348 (12.9)
Just completed high school	450 (16.7)
Diploma	279 (10.3)
Bachelor’s degree	522 (19.3)
Master’s degree	45 (1.7)
PhD’s degree	53 (2.0)
Income Level	
Less than 5,000 SR	722 (26.7)
Between 5,001 to 8000 SR	427 (15.8)
Between 8,001 to 11,000 SR	284 (10.5)
Between 11,001 to 13,000 SR	198 (7.3)
Between 13,001 to 16,000 SR	222 (8.2)
Between 16,001 to 20,000 SR	190 (7.0)
More than 20,000SR	151 (5.6)
No stable income	508 (18.8)

Table 2 highlights the prevalence of chronic diseases and mental health conditions among the older participants. Nearly half (44.7%) were diagnosed with hypertension and type 2 diabetes, while 59.5% had hypercholesterolemia. Furthermore, 52% of participants reported having two or more comorbidities, with 32.1% having three or more. Chronic respiratory diseases (12.0%) and heart disease (19.2%) were also common. Mental health risks were notable, with 17.7% at risk for depression and anxiety.

**TABLE 2:** Prevalence of chronic diseases and mental health among elderly participants.

Hypertension	
Yes	1,209 (44.7)
No	1,493 (55.3)
Hypercholesterolemia	
Yes	1,608 (59.5)
No	1,094 (40.5)
Type 2 Diabetes	
Yes	1,207 (44.7)
No	1,495 (55.3)
Liver disease	
Yes	182 (6.7)
No	2,520 (93.3)
Heart disease	
Yes	518 (19.2)
No	2,184 (80.8)
Stroke	
Yes	183 (6.8)
No	2,519 (93.2)
Cancer	
Yes	118 (4.4)
No	2,584 (95.6)
Chronic respiratory disease	
Yes	325 (12.0)
No	2,377 (88.0)
Genetic diseases	
Yes	243 (9.0)
No	2,459 (91.0)
Comorbidities	
Never	767 (28.4)
Signal disease	530 (19.6)
Two	538 (19.9)
Three and more	867 (32.1)
Depression	
At risk	479 (17.7)
Not at risk	2,223 (82.3)
Anxiety*	
At risk	391 (17.7)
Not at risk	1,817 (82.3)

Table 3 reveals key behavioral risk factors among older participants. A significant majority (88.9%) reported inadequate fruit and vegetable intake, while only 20.1% met physical activity recommendations. Obesity and overweight were prevalent, affecting 30.7% and 36.4% of participants, respectively. Regarding smoking habits, 11.1% reported daily cigarette smoking, while daily use of waterpipes and e-cigarettes was 5.8% and 5.0%, respectively. These findings emphasize the high prevalence of behavioral and lifestyle risk factors in this population.

**TABLE 3:** Risk factors among elderly participants.

Characteristics	Total (%)
Fruit and vegetable intake	
Acceptable fruits and vegetable level. (At least one portion of fruit and one portion of vegetables)	300 (11.1)
Low level of fruits and vegetable	2,402 (88.9)
Physical activity	
Physical activity as recommended (at least 150 min of moderate intensity activity a week or 75 min of vigorous intensity activity a week)	544 (20.1)
Low level of physical activity (Not meet the recommendation)	2,158 (79.9)
BMI	
Underweight	71 (2.6)
Normal	818 (30.3)
Overweight	983 (36.4)
Obese	830 (30.7)
Self-weighting	
This week	417 (15.4)
Last 2 weeks	491 (18.2)
Last month	584 (21.6)
More than month	1,210 (44.8)
Cigarette Smoking	
Never	2,253 (83.4)
Yes, daily	299 (11.1)
Yes, occasionally	150 (5.6)
Waterpipes smoking	
Never	2,411 (89.2)
Yes, daily	156 (5.8)
Yes, occasionally	135 (5.0)
E-Cigarette smoking	
Never	2,472 (91.5)
Yes, daily	134 (5.0)
Yes, occasionally	96 (3.6)

## 4 Discussion

This study explored the demographic characteristics, socioeconomic status, health conditions, and lifestyle behaviors of the older population in Saudi Arabia during 2022–2023. The findings revealed that 77% of the older people in the sample had lower education levels (less than a bachelor’s degree). Additionally, 52% of the sample had two or more chronic conditions. The assessment of mental health risks indicated that the risk of both anxiety and depression was approximately 17%. Most notably, this study found very low adherence to healthy behaviors and lifestyles, including low levels of healthy diet and physical activity, alongside high levels of obesity and cigarette smoking.

The lower educational level among older populations is a global challenge (Organisation for Economic Co-operation and Development OECD, 2022). For instance, in the United States, only 33% of the older population hold a bachelor’s degree or higher (Ryan and Bauman, 2016). In China, studies have shown that only about 3%–10% of older individuals in the sample have a bachelor’s degree or higher (Liu et al., 2022). In the Middle East, a study focusing on social participation among community-dwelling older adults in Iran found that lower educational levels were prevalent and significantly impacted social engagement (Shakeri et al., 2023). This highlights the need to tailor health interventions and programs to align with the educational profile of the older population in Saudi Arabia.

The prevalence of multimorbidity in Saudi Arabia, as indicated by this study’s results, is approximately similar to global figures (6). For instance, the prevalence of multimorbidity is 51% in Australia, 71% in Kuwait, and 52.8% in the United Kingdom (Kabir et al., 2022; Saoud et al., 2024; Kingston et al., 2018). systematic review reported that the prevalence of multimorbidity in the Middle East is approximately 21.8%, with variations across different countries (Alsuliman et al., 2022) The rising levels of multimorbidity have a significant impact on quality of life and healthy longevity. One systematic review found that for each additional chronic disease, quality of life decreases by 4.37% (Makovski et al., 2019). Another systematic review investigating the association between multimorbidity and mortality in older adults found that, compared to individuals without multimorbidity, the risk of death was 1.73 (95% CI: 1.41; 2.13) for those with two or more morbidities, and 2.72 (95% CI: 1.81; 4.08) for those with three or more morbidities (Nunes et al., 2016). The negative effects on quality of life and mortality may also be exacerbated by the presence of mental health risks. Furthermore, the impact on quality of life can extend to caregivers and family members (Jika et al., 2021). Therefore, it is crucial to continuously monitor the prevalence of multimorbidity among older people in Saudi Arabia and strategically plan to reduce it by focusing more on chronic disease prevention.

This study highlighted that the prevalence of obesity and overweight among older people in Saudi Arabia is nearly 67%. This high prevalence is accompanied by very low levels of fruit and vegetable consumption and low physical activity. The combination of these factors could exacerbate the issue of multimorbidity and significantly contribute to the further decline in quality of life and increased morbidity. Currently, there are no national studies specifically exploring the quality of life among the older population in Saudi Arabia. However, a study conducted in a primary care setting in one region of Saudi Arabia found that the quality of life among older individuals is suboptimal (Alqahtani et al., 2022). Therefore, it is crucial to accelerate the development of healthy behavioral programs focused on the older population, considering their low educational levels, to effectively maintain and improve national longevity targets.

## 5 Conclusion

This study provides a comprehensive profile of the older population in Saudi Arabia during 2022–2023, highlighting significant health, socioeconomic, and behavioral challenges. The findings indicate that the majority of the older population has low educational attainment, which may impede their ability to adopt health-promoting behaviors. Moreover, the high prevalence of chronic diseases and multimorbidity, coupled with low levels of physical activity and poor dietary habits, underscores the urgent need for targeted interventions to enhance the quality of life and health outcomes for this vulnerable group. The results also highlight the necessity of integrating mental health support into older care programs, given the substantial risk of depression and anxiety within this population. Moving forward, it is crucial for policymakers and healthcare providers in Saudi Arabia to develop and implement tailored health interventions that address the unique needs of older people, with a focus on preventive measures, education, and support systems. This approach will be vital in achieving the nation’s ambitious goals for healthy aging as outlined in Saudi Arabia’s Vision 2030.

## 6 Limitations and future research

This study provides a comprehensive descriptive profile of Saudi Arabia’s aging population; however, it is not without limitations. The descriptive design of this study inherently limits the ability to draw causal inferences or explore relationships between variables. While subgroup analyses and contrast statistics would have enriched the findings and allowed for a more detailed understanding of population differences, they were beyond the scope of this study, which aimed to establish baseline data for older people in Saudi Arabia.

Future research should build upon these findings by incorporating advanced statistical analyses, such as subgroup comparisons and multivariate models, to identify patterns and relationships among demographic, health, and behavioral variables. Such analyses can help uncover factors contributing to disparities in health outcomes and inform targeted interventions. Additionally, longitudinal studies would be valuable to track changes over time and assess the effectiveness of interventions tailored to the aging population.

Despite these limitations, the descriptive nature of this study serves as a critical foundation for understanding the demographic and health profiles of older adults in Saudi Arabia. By addressing the current knowledge gaps, this study paves the way for hypothesis-driven research and evidence-based policy development aimed at improving the health and quality of life of this growing population.

## Data Availability

The raw data supporting the conclusions of this article are available from Sharik Association for Health Research upon request.
